# Immune cells in thyroid adenoma and carcinoma: uncovering a hidden value of assessing tumor-host interplay and its potential application in thyroid cytopathology

**DOI:** 10.3389/fmolb.2025.1542821

**Published:** 2025-01-28

**Authors:** Iryna Omelianenko, Nazarii Kobyliak, Tetyana Falalyeyeva, Oleksii Seleznov, Pavlina Botsun, Lyudmila Ostapchenko, Oleksandr Korotkyi, Liudmyla Domylivska, Olena Tsyryuk, Galyna Mykhalchyshyn, Tetiana Shapochka, Oksana Sulaieva

**Affiliations:** ^1^ Medical Laboratory CSD, Pathology Department, Kyiv, Ukraine; ^2^ Educational-Scientific Center “Institute of Biology and Medicine” Taras Shevchenko National University of Kyiv, Kyiv, Ukraine; ^3^ Endocrinology Department, Bogomolets National Medical University, Kyiv, Ukraine; ^4^ Pathology Department, Kyiv Medical University, Kyiv, Ukraine

**Keywords:** thyroid cancer, immune cells, immune microenvironment, thyroid adenoma, papilary thyroid carcinoma

## Abstract

**Introduction:**

Although the role of tumor immune microenvironment (TIME) in thyroid cancer is well established, little data exists about the differences in immune cell presence in thyroid adenomas and carcinomas. We assume that immune cell density could be an additional diagnostic criterion for differentiating benign and malignant tumors in thyroid aspirates.

**Aim:**

The current study compared the immune contexture of thyroid adenoma (TA) and thyroid carcinoma (TC) in histological and cytological specimens of III-V categories.

**Materials and methods:**

This pilot study included 72 cases (36 of TA and 36 of TC) with verified histological diagnosis and pre-operative cytology corresponding to categories III, IV and V according to the Bethesda system for reporting thyroid cytology. The number of CD8+, CD68+ and CD163+ cells was assessed in histological samples of TA and TC with further comparison to cytological specimens. Besides, the expression of STAT6 and SMAD4 as potential regulators of TIME was evaluated in the study.

**Results:**

TC demonstrated an immune-rich profile representing abundant tumor-associated CD8+ lymphocytes, CD68 and CD163+ macrophages. In contrast, TA represented mostly a low immune cell infiltration. The higher immunogenicity of TC was accompanied by the more profound expression of STAT6 and SMAD4 in tumor cells. The number of immune cells in cytological specimens correlated with CD8+ (r = 0.693; p < 0.001) and CD163+ cells (r = 0.559; p < 0.001) in histological samples, reflecting the differences in the tumor immune microenvironment between benign and malignant thyroid neoplasms.

**Conclusion:**

TC demonstrated high immunogenicity compared to TA, which correlated to the number of immune cells in cytological specimens. The number of immune cells in thyroid cytology samples could be an additional criterion in cytological diagnostics for III-V Bethesda categories. Further investigations are needed to validate the findings of the study.

## Introduction

Thyroid cancer (TC) is one of the most common endocrine malignancies (Siegel et al., 2021). The incidence of TC morbidity has been increasing for the last decades leading to a sharp increase in thyroid surgery, the need for lifelong hormone replacement therapy and disturbances of endocrine regulation and metabolism (Rahib et al., 2014; Lim et al., 2017). Among the various types of TC papillary thyroid cancer prevails, while follicular, medullary and anaplastic carcinoma represents a smaller proportion of thyroid malignancies. Genetic and epigenetic research advances have identified the differences in mutagenesis and signaling pathway alterations in different thyroid cancers. It was shown that various histological types of TC are rooted in distinct molecular alterations and possess different evolution (Xing, 2013). These findings were incorporated into diagnostic algorithms relying on molecular testing applications in the case of “grey zone” categories of cytological diagnostics of thyroid fine needle aspiration biopsy (FNA) (Ali et al., 2023a). Although molecular testing provides a valuable impact on distinguishing benign and malignant tumors of the thyroid, its availability and affordability are limited especially in low- and middle-income countries. Seeking alternative approaches for discerning benign and malignant thyroid neoplasia shifted researchers’ attention toward the tumor immune microenvironment (TIME).

TIME is considered to be one of the crucial factors influencing the development and progression of malignant tumors. It comprises different types of immune cells, signaling molecules, and growth factors (Yang et al., 2021). TIME discovery has illuminated the bidirectional and multivariable interplay between tumor cells and the host immune system, defining the formation of conditions for malignancy and progression of tumor growth (Ferrari et al., 2019). The link between thyroid carcinoma and inflammation has been reported in many studies (Zheng et al., 2024; Wang et al., 2022; Li et al., 2024), uncovering both mechanisms of immune-mediated tumor destruction and tumor cell-dictated immune evasion (Menicali et al., 2021). However, little is known about the differences in immune cells’ presence in TA. These data could be essential for developing a new approach for differentiating benign and malignant thyroid tumors at the pre-operative stage that is based on assessing cytological features of FNA biopsy of thyroid nodules (Goldstein et al., 2002). Although FNA is approved as the best diagnostic approach for primary cytological assessment of thyroid nodules using the Bethesda system, it is still challenging to discriminate between benign and malignant thyroid tumors (Sulaieva et al., 2019). About 10%–20% of lesions, diagnosed by FNA as categories III (atypia of undetermined significance; AUS), IV (follicular neoplasm; FN) or V (suspicious for malignancy (SFM) still represent some uncertainty and require molecular testing for further patients management (Ali et al., 2023b). Although molecular evolution and profile of thyroid neoplasms are well discovered and various types of molecular testing were validated and approved for clinical practice, the high costs of molecular testing reduce its wide application, especially in low-resource settings (Fagin and Nikiforov, 2023). Beyond various ancillary tests focused on tumor cell biomarkers, signaling and genomics, assessment of TIME could be beneficial, as it reflects host-tumor interplay and its nature.

A complex interplay between various immune cells infiltrating thyroid carcinomas defines the balance between protumor and antitumor effects, impacting tumor cells’ behavior and prognosis (Li et al., 2024). Previous studies uncovered a prognostic role of CD8+ T-cells in papillary thyroid carcinoma (PTC) prognosis (Galdiero et al., 2016). Naturally, the functioning of CD8+ lymphocytes responsible for cell-mediated immunity was associated with antitumor defense and efficiency in killing tumor cells (Giles et al., 2023). Other studies addressed the involvement of CD4+ cells and B lymphocytes in PTC development and progression (Menicali et al., 2021; French et al., 2010). Tumor-associated macrophages (TAMs), including both M1 (CD68+) and M2-type (CD163+), were shown to be the most numerous immune cells infiltrating thyroid carcinoma (Liu et al., 2022). The impact of CD163+ tumor-associated macrophages representing the M2-anti-inflammatory type includes the production of a wide spectrum of growth factors, promoting cancer growth (Song et al., 2023). Their amount was linked to various clinicopathological features and positively correlated with larger tumor size, invasiveness and decreased survival in PTC (Gong et al., 2021; Jung et al., 2015). However, the precise mechanisms defining the scale and polarization of immune cells response within different thyroid tumors still need clarification.

Numerous studies addressed factors affecting the immune contexture of TC. In addition to various cytokines and chemokines, regulatory molecules involved in their signaling cascades have been placed at the center of studies. For instance, the recent study defined the prognostic role of the transcription factor of signal transducer and activator of transcription (STAT) family–STAT6, in thyroid cancer (Wang et al., 2020). In addition to the cell cycle, cell adhesion and apoptosis control STAT6 can impact immune infiltration of B cells, CD4+ T cells, neutrophils, macrophages, and dendritic cells. STAT6 also affects macrophage polarization due to IL-4 and IL-13 effects mediated through STAT6 signaling. Besides activated macrophages secrete transforming growth factor β (TGF-β) which also can affect immune cells response and TIME through SMAD4 signaling pathways (Wang et al., 2020). Although the role of STAT6 and SMAD4 is in the focus of active discovery there are still little data about the role of these molecules in TA and TC (Wang et al., 2020).

Although the immune microenvironment of thyroid malignancies is quite well established, it is still little known about the difference between the immune contexture of thyroid adenoma and carcinoma. This pilot case-control study aimed to compare the TIME of thyroid adenomas and carcinomas with respect to the potential mechanisms impacting tumor immune contexture and the correlation between histological and cytological representation.

## Materials and methods

### Ethics statement

This case-control study was approved by the Ethics Commission of the Medical Laboratory CSD (Protocol No. 1D from 02.10.2023). It was performed following the principles of the Declaration of Helsinki. The Ethics Commission of the Medical Laboratory CSD waived informed consent.

### Patients’ characteristics

**FIGURE 1:**
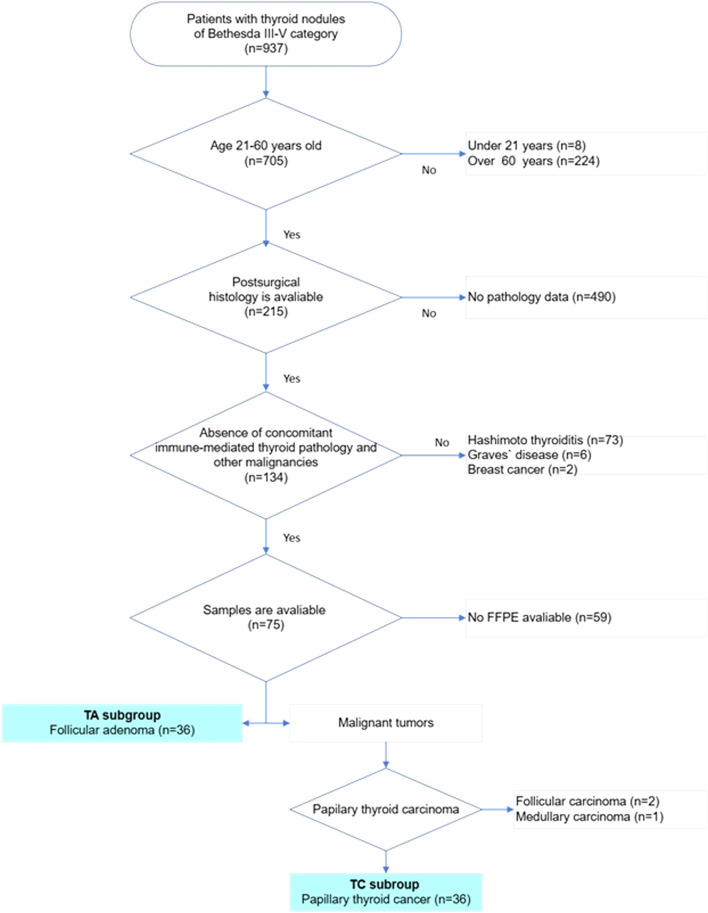
Flowchart of patients’ selection in the study. The multistep selection algorithm was applied in the study. After selecting cases in which thyroid cytology corresponded to categories III, IV or V according to the Bethesda system for reporting thyroid cytology, patients were checked for age. Only patients of age 21–60 were included in the study. Next, all cases were checked for follow-up surgery and a histopathology report was available. Cases with complete pathology reports were reviewed and all cases with reported autoimmune thyroid diseases were excluded. After that cases with available FFPE blocks were revised for diagnosis, 36 cases with follicular adenoma and 36 cases with papillary thyroid carcinoma were included in the final study sample.

72 cases of III-V categories of the Bethesda system and histologically confirmed diagnoses of TC (group 1, 36 cases, 12 cases of each Bethesda category) and TA (group 2, 36 cases, 12 cases of each Bethesda category) were selected for the study (Figure 1). All enrolled patients met the following criteria: 1) thyroid cytology corresponded to Bethesda categories III, IV or V; 2) histologically confirmed diagnosis of thyroid neoplasia, 3) age between 21 and 60 years, and 4) patients had no autoimmune thyroid diseases (Grave`s disease or Hashimoto`s thyroiditis); 5) absence of other malignancies at the time of examination. As far as PTC was the most common type of thyroid malignancy, only individuals with PTC were included in this study. Exclusion criteria were as follows: 1) age under 21 or over 60; 2) Bethesda category I, II, or VI at cytology; 3) patients having any co-existing immune-mediated thyroid pathology (Hashimoto thyroiditis, Graves’ disease, etc.); 4) other histological types of TC (follicular thyroid carcinoma or medullary thyroid carcinoma); 5) individuals having synchronous or metachronous malignancies.

The age of patients with TA was 37 (IQR 29–53) and the age of TC comprised 42.5 (IQR 27–54). Other clinical and demographic data are represented in Table 1.

**TABLE 1:** Clinic pathological characteristics of patients.

Patients characteristics	TA group	TC group
Number of patients	**36**	**36**
Sex		
Females	34	35
Males	2	1
Age	37 (IQR 29–53)	42.5 (IQR 27–54)
Laterality of lesions		
Unilateral	36	35
Bilateral	0	1

the bold indicates significant changes.

### Histology and immunohistochemistry

The number of immune cells infiltrating thyroid tumors was assessed in histological specimens. Special attention was paid to T-lymphocytes (CD8^+^), B-lymphocytes (CD19^+^) and macrophages (CD163+ and CD68^+^). The expression of the signaling molecules STAT6 and SMAD4, which play a crucial role in regulating the immune response and the tumor microenvironment, was also analyzed. Evaluating the expression of these markers allows for a better understanding of the mechanisms of tumor interaction with the immune system and the role of immunosuppression in the progression of the tumor process.

Immunohistochemistry was applied to visualize different immune cells and evaluate STAT6 and SMAD4 expression (Table 2). Briefly, paraffin tissue sections with a thickness of 4 μm were prepared and placed on SuperFrost Plus slides (Menzel, Germany). Deparaffinization and restoration of antigenicity were performed in TRS High pH buffer (DAKO, Denmark) at a temperature of 98°C for 40 min in a DAKO PT Module apparatus. The following primary antibodies were used for staining: CD8 (clone C8/144B, DAKO), CD4 (clone 4B12, DAKO), CD19 (clone LE-CD19, DAKO), CD68 (clone KP1, DAKO), CD163 (clone MRQ-26, Cell Marque), STAT6 (clone EP325, Cell Marque), and SMAD4 (clone JM56, Novocastra). Appropriate detection systems were used with incubation of primary antibodies for 30 min and subsequent treatment with DAB solution.

**TABLE 2:** Characteristics of biomarkers used for immunohistochemistry.

Marker	Clone	Manufacturer	Interpretation
CD8	C8/144B	DAKO	A T-cell marker that detects cytotoxic/suppressor cells in lymphocytes; also found on NK-cells
CD68	KP1	DAKO	Lysosomal membrane glycoproteins expressed on monocyte-macrophage cells
CD163	MRQ-26	Cell Marque	Acute phase regulatory transmembrane protein that induces signaling and is found only in cells of monocytic origin
STAT6	EP325	Cell Marque	A transcription factor involved in signaling pathways associated with immune responses, including macrophage polarization and Th2 cell differentiation; STAT6 is activated by cytokines such as IL-4 and IL-13
SMAD 4	JM56	Novocastra	A protein that plays an important role in the signaling pathway activated by TGF-β (transforming growth factor beta). It is a central mediator of signaling in this pathway and is involved in the regulation of cell growth, differentiation and apoptosis, and immune response and inflammation

Immune cells in histological slides were counted in 10 fields of view at ×40 magnification using light microscopy (Leica Microsystems, DM3000). The number of immunopositive cells was evaluated per square millimeter. In addition, the density of immune cell infiltration was assessed in a dichotomic way as high or low. The threshold for such discrimination was based on the Median (9 per 1 mm^2^ for CD8 lymphocytes and 17 per 1 mm^2^ for CD68^+^ and CD163^+^ macrophages).

Two independent cytologists assessed cytological specimens of the enrolled cases to verify the category according to the Bethesda system and estimate the number of immune cells (lymphocytes and macrophages). The number of immune cells in cytological slides was evaluated semi-quantitatively (0 – lack of immune cells, 1 – few immune cells, 2 – moderate number of immune cells and 3 – high number of immune cells).

In addition, the expression levels of STAT6 and SMAD4, being important factors involved in tumor signaling cascades, were evaluated (Table 2). Expression levels of these proteins were evaluated in both stromal and tumor cells. The expression of STAT6 and SMAD4 in tumor cells was evaluated semi-quantitatively using an intensity scale from 0 to 3 (0 - negative, 1 - weak, 2 - moderate, 3 -strong) and the percentage of positive cells (0% - negative, 1%–25% - 1+, 26%–50% - 2+, >50% - 3+) (Sulaieva et al., 2020a). The total score was calculated by multiplying the degree of staining by the percentage of positive cells, which allows us to estimate the relative degree of expression of these factors in the tumor tissue. A blind histologic analysis was performed by two independent pathologists.

### Statistical analysis

Statistical analysis was performed using MedCalc software (MedCalc Software, Mariakerke, Belgium) and GraphPad Prism, version 10.4.0 (GraphPad Software, Inc., La Jolla, CA, United States) for creating diagrams. The data distribution was analyzed using the Shapiro-Wilk test for normality. All continuous variables were presented as the Median with interquartile range (IQR), and categorical variables were presented as %. For comparing quantitative data between TA and TC Mann-Whitney test was applied. Categorical variables were analyzed by utilizing a χ^2^ test. Spearman’s correlation analysis was used to assess associations between different immune cells and biomarker expression. p-value ≤0.05 was considered statistically significant.

## Results

### Immune cells in TA and TC

**FIGURE 2:**
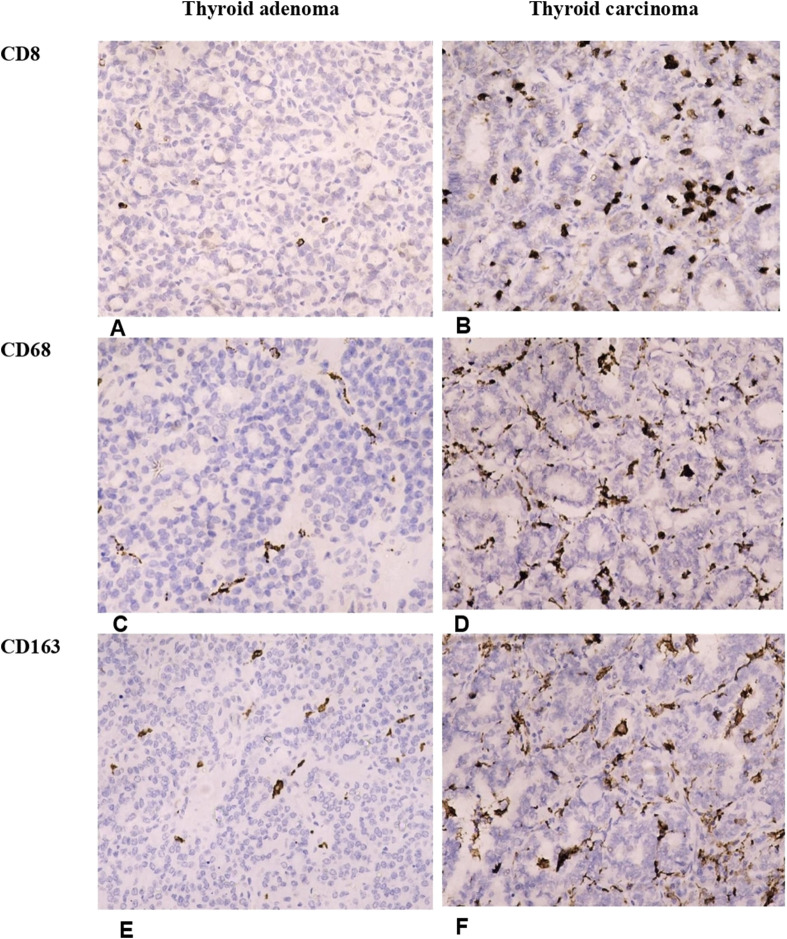
Differences in the number of T-cytotoxic lymphocytes and macrophages in thyroid adenomas and papillary carcinomas. Thyroid adenoma demonstrates a low number of tumor-infiltrating lymphocytes and macrophages. In contrast, the immune cells were significantly more numerous in thyroid carcinoma. **(A, B)** Illustrate the number of CD8+ T-cytotoxic lymphocytes in follicular adenoma **(A)** and papillary thyroid carcinoma **(B)**. **(C, D)** Represent infiltration of follicular adenoma **(C)** and papillary thyroid carcinoma **(D)** by CD68+ macrophages. **(E, F)** Show the density of tumor-infiltrating M2-macrophages in follicular adenoma **(E)** and papillary thyroid carcinoma **(F)**. Immunohistochemistry. Magnification 200.

**FIGURE 3:**
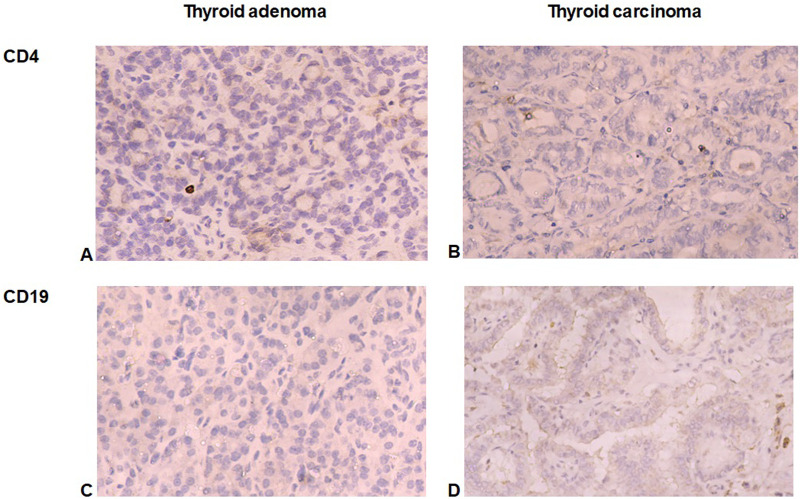
CD4+ and CD19+ cells in thyroid tumors. **(A, B)** Demonstrate a little number of CD4+ cells in follicular adenoma **(A)** and papillary thyroid carcinoma **(B)**. **(C, D)** Illuminate scarce CD19+ B-lymphocytes in follicular adenoma **(C)** and papillary thyroid carcinoma **(D)**. Immunohistochemistry. Magnification 200.

The immune microenvironment of TC was enriched with immune cells with the prevalence of tumor-associated lymphocytes and macrophages. CD8+ lymphocyte number was significantly higher in TC compared to TA (p < 0.001, Figure 2). Surprisingly, only scarce CD4+ and CD19+ cells (from 0 to 3 cells per 1 mm^2^) were found in samples of TC and TA (Figure 3).

Likewise, CD8+ cells, the number of tumor-associated macrophages was significantly higher in carcinomas (p < 0.001). CD68+ and CD163+ cells were abundant in TC forming a network within the tumors (Figure 2; Table 3). Notably, the density of infiltration by M1 and M2 macrophages displayed a high heterogeneity in patients of both groups. Although the Medians of CD68+ and CD163+ cells in the TC group were almost similar, the balance of M1 and M2 macrophages varied significantly inside the group, so the CD68/CD163 ratio was low in patients with papillary thyroid carcinoma, comprising 0.563 (0.082–3.0). In contrast, in TA the proportion of CD68+ to CD163+ cells was significantly higher (p = 0.036) reaching 1.04 (0.467–3.0).

**TABLE 3:** Characteristics of immune contexture of TA and TC.

Immune cells	TC	TA	p
Immune cells count within the tumor			
CD8	6 (1.2–19.5)	37 (13.5–77)	p < 0.001
CD68	9 (4–15)	21 (6–46)	p < 0.001
CD163	7 (2–12.5)	21 (4.5–45)	p < 0.001
CD68/CD163 ratio	0.563 (0.082–3.0)	1.04 (0.467–3.0)	p = 0.036
IHC score of transcription factors expression			
STAT6	1 (0–1.5)	3 (1–6)	p = 0.008
SMAD4	3.5 (1–6)	6 (1–6)	p = 0.026

The data are presented as the Me (Q_I_ - Q_III_).

**FIGURE 4:**
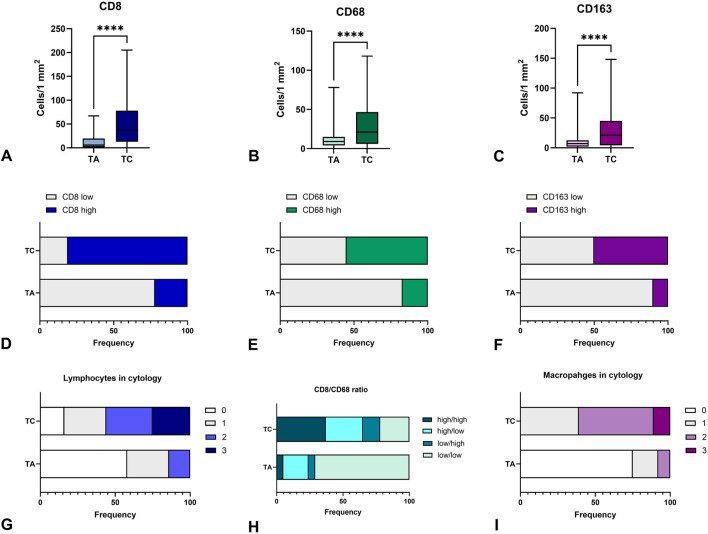
Immunogenicity of thyroid carcinomas and adenomas. **(A–C)** – demonstrate the number of CD8+, CD68+, and CD163+ cells per 1 mm^2^ in thyroid tumors, respectively. TC demonstrated a higher number of CD8+ T-cells, CD68+ and CD163 macrophages infiltrating the tumor as compared to TA. **(D–F)** – represent the shares of cases with low or high numbers of CD8+, CD68+ and CD163+ cells in thyroid tumors, respectively. More than half of TC cases demonstrated high infiltration by immune cells. **(G)** - demonstrates the proportion of cases with different lymphocyte numbers in FNA cytology of confirmed TA and TC, where 0 corresponds to the lack of lymphocytes, 1, 2 and 3 demonstrate categories of mild, moderate and high numbers of lymphocytes in cytological specimens. More than half of TC cases showed a high or moderate number of lymphocytes in cytological specimens. **(H)** - represents the proportion of cases with different CD8+/CD163+ cell ratio. **(I)** - demonstrates the proportion of cases with different macrophage numbers in FNA cytology of confirmed TA and TC, where 0 corresponds to the lack of lymphocytes, 1, 2 and 3 demonstrate categories of mild, moderate and high numbers of macrophages in cytological specimens. More than half of TC cases showed a high or moderate number of macrophages in cytological specimens. TA – thyroid adenoma, TC – thyroid adenoma.

When applying dichotomic assessment of immune cell numbers as low or high in thyroid tumors, we found that most TA demonstrated a low number of CD8+, CD68+ and CD163 cells. Alternatively, about 80% of TC demonstrated high immunogenicity, being “hot” in terms of T-cell infiltration, and more than half of them possessed a high number of macrophages (Figures 4A–F). These facts reflect a higher immunogenicity of thyroid carcinoma as compared to benign tumors.

For uncovering the potential mechanisms affecting tumor immune contexture, at the next step, we assessed the expression of signaling biomarkers involved in carcinogenesis and immune modulation.

### STAT6 and SMAD4 expression in TA and TC: relation to diagnosis and immune cell number

**FIGURE 5:**
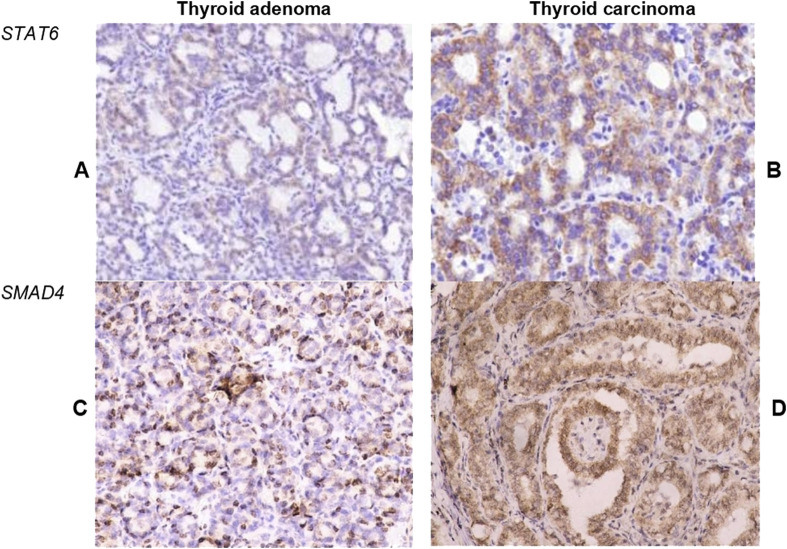
Expression of STAT6 and SMAD4 in thyroid tumors. **(A, B)** Demonstrate STAT6 expression in tumor cells within follicular adenoma **(A)** and papillary thyroid carcinoma **(B)**. TC demonstrated significantly higher intensity and extension of STAT6 expression. **(C, D)** Illustrate the expression of SMAD4 in cells of follicular adenoma **(C)** and papillary thyroid carcinoma **(D)**. Most cells of TC demonstrated moderate to high nuclear and cytoplasmic expression of SMAD4. Immunohistochemistry. Magnification 200.

Differences in immune cell count between benign and malignant thyroid tumors were also associated with distinction in STAT6 and SMAD4 expression (Figure 5). A significantly higher expression of STAT6 s SMAD4 was found in TC, compared to TA. In adenomas, about half of tumor cells showed mild cytoplasmic expression of STAT6, whereas in TC STAT6 demonstrated moderate expression in a higher proportion of tumor cells (p = 0.008) (Table 3). STAT6 score in TC moderately correlated with the number of CD163+ macrophages (r = 0.394; p < 0.001) and CD68^+^ cells (r = 0.317; p < 0.001).

Nuclear expression of SMAD4 was detected in less than half of tumor cells in TA but showed diffuse moderate expression in carcinoma cells (Figure 5). Interestingly, SMAD4 expression correlated with STAT6 (r = 0.495, p < 0.001) and was also linked to the number of CD163+ cells (r = 0.41; p < 0.001) in TC.

### Correlations between cytological and histological features of thyroid tumors immune contexture

Similar to histological samples, cytological specimens demonstrated significant differences in lymphocyte and macrophage numbers between cases diagnosed as TA and TC. The proportion of cases with high and moderate numbers of lymphocytes and macrophages was significantly higher in TC compared to TA (p < 0.001). While most TA cases demonstrated a lack or low number of macrophages, cases verified as TC were more commonly rich in macrophages (Figure 4G) and/or lymphocytes (Figure 4I).

The number of macrophages in cytological specimens correlated with the amount of CD163+ cells in histological samples (r = 0.559; p < 0.001). Similarly, lymphocyte density in cytological slides correlated to the amount of C8+ cells (r = 0.693; p < 0.001) although we did not find a correlation between the number of immune cells in FNA specimens and the expression of STAT6 and SMAD4 in histological samples.

To illustrate the relationship of thyroid tumors’ immune contexture in cytological and histological specimens, herein we provide several examples.

Example 1 A woman, 50 years old, single thyroid nodule 2 cm in diameter, Bethesda category 3 with architectural or nuclear atypia at cytology. Cytological specimens demonstrated a low number of lymphocytes and only a few macrophages. After repetitive FNA, the results of cytology were the same. According to the patient`s preferences, a lobectomy was performed. Follicular adenoma was diagnosed after a histopathological study. Assessment of tumor immunogenicity by immunohistochemistry revealed the following number of immune cells: CD8+ cells - 11 per 1 mm^2^, CD68+ – 6 per 1 mm^2^, CD163+ macrophages – 3 per 1 mm^2^. The ratio of CD68/CD163 macrophages comprised 2. STAT6 score was 2, and SMAD4 score reached 2.

Example 2 A woman, 54 years old, unilateral thyroid nodule 2 cm in size, Bethesda class 3 with architectural and nuclear atypia. There was a moderate number of macrophages and a mild number of lymphocytes in cytological specimens. After surgery, the histological diagnosis was Papillary thyroid carcinoma. Assessment of tumor immunogenicity by immunohistochemistry revealed the following number of immune cells: CD8+ cells - 15 per 1 mm^2^, CD68+ – 7 per 1 mm^2^, CD163+ macrophages – 35 per 1 mm^2^. The ratio of CD68/CD163 macrophages comprised 0,2. STAT6 score was 6, and SMAD4 score reached 4.

Thus, the number of immune cells in cytological specimens correlated with CD8^+^ and CD163^+^ cells in histological samples (Figure 4H) and can reflect the differences in the immune tumor microenvironment between benign and malignant thyroid tumors that could be applied as additional criteria in cytological diagnostics for III-V Bethesda categories.

## Discussion

The results of our research showed a significantly higher level of infiltration of TC by tumor-infiltrating CD8+ lymphocytes and tumor-associated macrophages compared to adenomas, which confirms the role of the immune microenvironment in the development and progression of papillary thyroid carcinoma (PTC). In particular, we found that the majority of immune cells in carcinomas are CD8+ cells, the number of which was more than three times greater in TC than in TA. This reflects a higher immunogenicity of carcinomas compared to benign tumors. CD8+ cytotoxic T-lymphocytes are well-known effector cells of cell-mediated immunity, are responsible for the elimination of tumor cells (Huang et al., 2019). Research by Modi et al., found that patients with PTC who had dense CD8^+^ T-cell infiltration showed slower tumor progression, smaller tumor sizes, and lower recurrence rates (Modi et al., 2003). This emphasizes the importance of the immune response mediated by cytotoxic T-cells in suppressing the tumor process and improving the prognosis for patients. This is consistent with previous studies indicating an important role of CD8^+^ T-cells in the antitumor response and regulating the immune microenvironment in various cancers, including thyroid cancer (Giles et al., 2023), although some studies highlighted the possible role of CD8+ in prognosticating TC recurrence (Cunha et al., 2015). It is also important to note that CD8+ cells were the most numerous immune cells in TC, prevailing under B-cells and tumor-associated macrophages that can explain the relatively slow tumor progression, satisfactory prognosis and high survival rate of papillary thyroid cancer, the most common histological type of TC (Sheridan et al., 1995; Ulisse et al., 2021). In this study we found only scarce CD4+ and CD19+ cells, reflecting a weak role of T-helpers and humoral immunity cells in thyroid tumors microenvironment. This surprising finding could be related to the exclusion of patients with coexisting thyroiditis from the study. As it was shown in recent studies, autoreactive CD4+ T cells and CD8+ cytotoxic T cells, as well as plasma cells producing autoantibodies play a central role in Hashimoto thyroiditis, which is often associated with thyroid tumor development (Chistiakov, 2005). However, in the absence of concomitant immune-mediated thyroid pathology, the role of CD4+ and CD19+ cells is much less significant. At the same time, it is worth noting that in this study we did not focus on discovering the role of various CD4+ cell and B-lymphocyte subsets, but rather explored the core differences of immune contexture of benign and malignant tumors of the thyroid and their correlation to cytological features of FNA (Chistiakov, 2005).

Of particular interest is the increased number of CD163+ macrophages in carcinomas compared to adenomas. Tumor-associated macrophages in the tumor microenvironment express a number of markers, such as CD163, indicating an M2-like polarization state of macrophages with tumor-protective functions (Aras and Zaidi, 2017; Sulaieva et al., 2020b). We found that the number of CD163+ cells in carcinomas was three times higher than in adenomas, confirming the role of these cells in the progression of PCOS. This trend of increased presence of M2 macrophages correlates with the data of other studies, which indicate that they will prevail in the tumor microenvironment (Sari et al., 2022). Moreover, the CD68/CD163 ratio was significantly lower in the TC group demonstrating the prevalence of anti-inflammatory M2-macrophages producing a wide range of angiogenic and tumor-promoting growth factors, cytokines and chemokines. In contrast to M1-macrophages, producing proinflammatory cytokines (tumor necrosis factor-alpha - TNF-α, IL-1β, IL-12, and IL-23) reactive oxygen species and NO, medicating inflammatory reaction with activating tumor-killing mechanisms (Chen et al., 2023), M2-macrophages contribute to cell dedifferentiation, tumor growth promotion, angiogenesis and enhanced invasiveness of cancer cells (Liu et al., 2022). These effects are rooted in the extensive secretion of Wnt1 and Wnt3 ligands, inducing activation of β-catenin activation, growth factors (VEGF, IGF, EGF, etc.) and anti-inflammatory cytokines (IL-6, IL-10, IL-18, TGFβ1 etc.) (Lv et al., 2021; Zhang et al., 2021).

The correlation between CD163+ cells and CD8+ (r = 0.395) may indicate the relationship between innate and adaptive immunity reactions during tumor evolution. Significant differences in the number of immune cells of innate and adaptive immunity allow generating the hypothesis that the difference in the immune context of benign and malignant thyroid tumors and carcinomas should be taken into account at the stage of cytological diagnosis of FNA. However, due to the limitations of our study (small sample size and lack of long-term follow-up), there is a need for further thorough research to test this hypothesis and to define clear criteria that will allow differentiation of different thyroid tumors taking into account immune cells in thyroid cytopathology practice.

Our results demonstrate that STAT6 and SMAD4 molecules are interconnected with the number of certain classes of immune cells, and therefore may play a role in the regulation of the immune microenvironment of thyroid tumors. STAT6 demonstrated a moderate positive correlation with cytotoxic T-lymphocytes (CD8+) and M2-macrophages (CD163+), which reflects the involvement of this transcription factor in the regulation of not only adaptive immune reactions but also the modulation of the macrophage phenotype towards the M2-phenotype, which promotes immunosuppression and tumor growth (Chen et al., 2023). The unique role of STAT6 in the polarization of macrophages to the M2 phenotype, which is associated with tumor progression, should be emphasized. STAT6 impacts the function of immune cells by inducing the transcription of genes involved in cell-mediated and humoral immunity (Hebenstreit et al., 2006). Typically, STAT6 stimulation occurs in response to IL-4 and IL-13 receptor binding and JAK1/JAK3 activation. Enhanced STAT6 expression in immune cells defines the polarization of macrophages toward M2-type (Chen et al., 2023). However, over the last decades, the elevated expression of STAT6 was found in various cancers. Researchers argue that STAT6 might play a prominent role in tumorigenesis and malignant transformation (Todaro et al., 2008; Wang et al., 2010). Beyond immune reaction, STAT6 can orchestrate cancer cell proliferation and apoptosis, cell adhesion and invasiveness, chromatin compaction and DNA damage response (Liu et al., 2017). STAT6 expression in tumor cells can impact the formation and composition of the tumor microenvironment facilitating immune cell recruitment and macrophage polarization (Sulaieva et al., 2020a).

In this study, we also found that TC demonstrated much higher expression of SMAD4 in tumor cells compared to TA. SMAD4 as a key signal transducer of TGFβ impacts a wide range of cellular processes, including proliferation, differentiation and apoptosis (D’Inzeo et al., 2012). Dysregulation of TGFβ signaling was shown to play an important role in tumor progression, affecting such processes as epithelial-mesenchymal transition, cell invasiveness and immune evasion mechanisms. Previous studies showed that reduction of SMAD4 may play a significant role in thyroid carcinogenesis, while overexpression of SMAD4, can facilitate antiproliferative response to TGFβ, reducing the invasive behavior of these cells (D’Inzeo et al., 2010). On the other hand, the recent discovery of the relations between SMAD4 expression and TIME defined the opposite results. Enhanced TGFβ secretion was illuminated in various malignancies, promoting cell invasiveness and tumor progression (Derynck et al., 2021). It was shown that TGF-β acting through SMAD4 and SMAD7 transduction enhances the recruitment of monocytes and activation of macrophages, suppresses the functioning of anti-tumor T-cells and generates immunosuppressive TIME facilitating immune evasion mechanisms and tumor progression (Derynck et al., 2021; Ivanova et al., 2018). These data are supported by the results of our study showing that SMAD4 expression in tumor cells correlated with the number of CD163+ macrophages. This reveals the potential role of the TGF-β-SMA4 signaling pathway in the malignancy-associated modulation of TIME and stimulates further discovery of the diagnostic and prognostic role of STAT6 and SMAD4 in thyroid tumors.

Finally, we found the correlation between cytological and histological reflections of tumor immunogenicity. Previous descriptive studies also highlighted the importance of assessing minor morphological features for navigating decisions in case of uncertain categories defined according to the Bethesda system (Rocha et al., 2023). In our study, we focused exclusively on TIME assuming that differences in benign and malignant tumors immunogenicity could be detected during thyroid FNA cytopathological review. Indeed, the assessment of hidden differences in tumor-host interplay allows rethinking the approach for cytological diagnostics shifting focus from follicular toward immune cells in challenging cases. Herein we showed that while most TA demonstrated a low level of immune cells, TC represented much more prominent immunogenicity, reflected in a number of immune cells in cytological samples. Cytological “grey zone” samples taken during FNA demonstrate significant differences in risk of malignancy and require further follow-up, molecular testing, or diagnostic lobectomy. Molecular testing, including Afirma Genomic Sequencing Classifier (GSC) and ThyroSeq v3, demonstrates high diagnostic performance and provides important prognostic data (Hu et al., 2022). It is informative for stratifying cytologically indeterminate FNA samples and defining high-risk patients with thyroid nodules. On the flip side, molecular testing is expensive, and its applications are not feasible in many countries due to high costs and technological barriers. This dictates the need for developing affordable and cost-effective approaches for managing cases of cytologically indeterminate thyroid nodules, and current data on immunogenetics of thyroid neoplasia could drive the novel approach based on considering immune microenvironment of thyroid lesions.

The findings of this pilot study demonstrate a hidden value of assessing immune cells in the case of follicular cell atypia and uncertainty of cytopathological conclusions.

### Limitations

The pilot study was conducted with limited sample size and did not count tumor stage, comorbid pathology and genetic alterations. Besides, this study did not cover the clinical data, cytokine profile and molecular testing results. The observations of this pilot study did not include Hashimoto thyroiditis, Graves’ disease and other immune-mediated pathologies of the thyroid gland. Taking into account the design of the study and limited sample size, further large-scale studies (preferably longitudinal investigations) for validating the approach and estimating the predictive values of assessing immune cells in FNA samples are needed.

## Conclusion

Thyroid carcinomas demonstrate high immunogenicity compared to adenomas with extensive infiltration by CD8+, CD68+ and CD163+ macrophages, associated with increased expression of SMAD4 and STAT6 in tumor cells. The number of immune cells in cytological specimens correlates with TILs and TAMs count in histological slides and can reflect the differences in the immune tumor microenvironment between benign and malignant thyroid tumors that could be applied as additional criteria in cytological diagnostics for III-V Bethesda categories.

## Data Availability

The raw data supporting the conclusions of this article will be made available by the authors, without undue reservation.
